# Evaluation of model fit in nonlinear multilevel structural equation modeling

**DOI:** 10.3389/fpsyg.2014.00181

**Published:** 2014-03-04

**Authors:** Karin Schermelleh-Engel, Martin Kerwer, Andreas G. Klein

**Affiliations:** Department of Psychology, Goethe UniversityFrankfurt, Germany

**Keywords:** multilevel structural equation modeling, interaction effect, level-specific model fit, likelihood ratio test, robust test statistic

## Abstract

Evaluating model fit in nonlinear multilevel structural equation models (MSEM) presents a challenge as no adequate test statistic is available. Nevertheless, using a product indicator approach a likelihood ratio test for linear models is provided which may also be useful for nonlinear MSEM. The main problem with nonlinear models is that product variables are non-normally distributed. Although robust test statistics have been developed for linear SEM to ensure valid results under the condition of non-normality, they have not yet been investigated for nonlinear MSEM. In a Monte Carlo study, the performance of the robust likelihood ratio test was investigated for models with single-level latent interaction effects using the unconstrained product indicator approach. As overall model fit evaluation has a potential limitation in detecting the lack of fit at a single level even for linear models, level-specific model fit evaluation was also investigated using partially saturated models. Four population models were considered: a model with interaction effects at both levels, an interaction effect at the within-group level, an interaction effect at the between-group level, and a model with no interaction effects at both levels. For these models the number of groups, predictor correlation, and model misspecification was varied. The results indicate that the robust test statistic performed sufficiently well. Advantages of level-specific model fit evaluation for the detection of model misfit are demonstrated.

## Introduction

Multilevel structural equation modeling (MSEM) has gained increasing attention over the last decades, as it combines advantages of multilevel modeling (MLM) and structural equation modeling (SEM) (cf. Muthén, 1994; Mehta and Neale, 2005; Hox et al., 2010). MLM has been developed for the analysis of clustered data and attempts to partition observed variances and covariances into within- and between-group components, while SEM aims at modeling the variances and covariances by taking the measurement errors into account. With the exception of cross-level interactions, MSEM generally incorporates linear relationships among latent variables at the within-level and at the between-level.

For the analysis of nonlinear single-level SEM with interaction or quadratic effects in the structural model, several methods have been developed (for an overview see, e.g., Schumacker and Marcoulides, 1998; Marsh et al., 2004; Klein and Muthén, 2007; Moosbrugger et al., 2009; Brandt et al., in press). These approaches include distribution-analytic approaches (Klein and Moosbrugger, 2000; Klein and Muthén, 2007), product indicator approaches (e.g., Jöreskog and Yang, 1996; Marsh et al., 2004; Little et al., 2006; Moosbrugger et al., 2009), Bayesian approaches (e.g., Lee et al., 2007; Song and Lu, 2010), and method of moment approaches (e.g., Wall and Amemiya, 2003; Mooijaart and Bentler, 2010; Brandt et al., in press). The most often used methods are the unconstrained product indicator approach (Marsh et al., 2004) and the latent moderated structural equations approach (LMS; Klein and Moosbrugger, 2000). For the analysis of nonlinear MSEM only these two approaches have already been applied.

The unconstrained product indicator approach has been developed for the estimation of latent interaction effects in single-level SEM with robust properties when distributional assumptions are violated. Products of indicator variables need to be constructed to identify the latent product (interaction or quadratic) terms. The parameters related to the measurement model of the latent nonlinear term are freely estimated, an advantage compared to the constrained approach which models these parameters as nonlinear functions of linear parameters (Jöreskog and Yang, 1996). For parameter estimation a maximum likelihood (ML) method developed for linear models is used which assumes multivariate normality of the indicator variables, an assumption violated in latent interaction models. Although parameter estimators are asymptotically unbiased standard errors are known to be generally underestimated (cf. Moosbrugger et al., 2009). A model test that takes the nonnormality induced by product indicators into account has not been developed yet, but a test statistic for linear models based on the comparison the empirical and the model-implied covariance matrix is available.

LMS is a distribution-analytic approach which does not require the forming of product indicators. Instead, LMS exploits the specific type of nonnormality implied by latent nonlinear effects for parameter estimation by using conditional distributions to represent the nonlinearity in the model (cf. Klein and Moosbrugger, 2000; Kelava et al., 2011). The nonnormal density function of the joint indicator vector is approximated by a finite mixture distribution of multivariate normally distributed components. For parameter estimation a ML method is used especially tailored for nonlinear SEM. LMS parameter estimators are therefore unbiased and highly efficient. A model test is not yet available as an adequate saturated model which in addition to the linear relations in the model also takes the nonlinearity induced by product terms into account has not been defined yet. A χ^2^ difference test based on likelihood values is provided for testing the significance of single model parameters. The power to detect nonlinear effects is higher for LMS than for the unconstrained approach.

Only recently researchers have started to investigate level-specific nonlinear effects (i.e., interaction or quadratic effects) in MSEM (Marsh et al., 2009; Leite and Zuo, 2011; Nagengast et al., 2013). Using the unconstrained approach, Nagengast et al. (2013) tested the expectancy-value model of motivation in a nonlinear MSEM and found a significant latent interaction effect between homework expectancy and homework value in predicting homework engagement at the within-group (student) level. Using LMS, Marsh et al. (2009) extended the tests of the big-fish-little-pond effect by investigating a latent quadratic effect of students' individual achievement and a latent interaction between gender and achievement on academic self-concept at the within-group level. However, these nonlinear effects did not reach statistical significance.

Up to now only a single simulation study for nonlinear MSEM exists using the unconstrained approach (Leite and Zuo, 2011). In this study, two types of mean centering, i.e., grand-mean centering (cf. Marsh et al., 2009) and residual centering (cf. Little et al., 2006), were applied for the analysis of a nonlinear MSEM with a single latent interaction effect at the between-group level. Results showed that both types of mean centering performed equally well for detecting the interaction effect when product indicators were highly reliable, while mean centering tended to perform slightly better for less reliable product indicators.

These few studies already indicate that single nonlinear effects can be detected using both approaches. However, researchers are generally interested in the overall fit of the nonlinear MSEM and not only in the significance of a single parameter. Unfortunately, the model fit cannot be determined as no adequate test statistic is provided by either of the nonlinear approaches. Researchers therefore investigate the model fit of a linear MSEM using the χ^2^ test before including the product term in the model (cf. Nagengast et al., 2013), although this practice is questionable because the assumptions of multivariate normality and homoscedastic residuals are violated for the linear model if there are nonlinear effects in the population model.

Evaluating the fit of nonlinear MSEM therefore presents a challenge. Although LMS does not provide any model test, the unconstrained product indicator approach nevertheless provides the likelihood ratio test developed for linear models. This test is based on the comparison of the unstructured and the model-implied covariance matrix with product terms included in the matrices as if they were observed variables. As the product variables are always nonnormally distributed, the overall model test does not follow a central χ^2^ distribution.

However, most statistical programs provide a robust test statistic corrected for nonnormality in the data (cf. Bentler and Dijkstra, 1985; Satorra and Bentler, 1994; Yuan and Bentler, 1998). Although the robust test statistic has originally been developed to correct the inflated test statistic due to unwanted nonnormality in the data, it may nevertheless correct the test statistic sufficiently well due to nonnormality resulting from products of normally distributed variables. In our simulation study the robust test statistic will therefore be used as if the normality assumption were just violated because of a multivariate nonnormality resulting from, e.g., floor or ceiling effects, rather than from the specific type of nonnormality implied by latent interactions.

The main goal of this study is to investigate the performance of the robust test statistic compared to the uncorrected ML test statistic for nonlinear MSEM using the unconstrained approach. In a Monte Carlo study we will investigate whether the robust test statistics is able to reliably detect misspecification of a nonlinear MSEM at the within-group level, at the between-group level, and at both levels simultaneously. Cross-level interaction, which occurs when the random slope of a within-group variable is predicted by a between-group level, will not be considered in this study, because this type of nonlinear effect poses a particular challenge for model fit evaluation. As level-specific model evaluation has been shown to be more informative for the detection at which level the misfit occurs (cf. Ryu and West, 2009; Ryu, 2011), we will also investigate the level-specific model fit.

## Nonlinear multilevel SEM

In the following, we will use the unconstrained product indicator approach for the analysis of a nonlinear MSEM with interaction effects at both levels. This approach needs the forming of product variables as indicators of the latent interaction terms. The nonlinear MSEM contains a covariance structure at each level, and components are needed in order to fit the model at the between and the within level.

If data are collected from *N* individuals (*i* = 1,…, *N*) nested in *J* groups (*j* = 1,…, *J*), the data vector ***y***_*ij*_ of subject *i* in group (cluster) *j* is decomposed into the sum of the group (cluster) average component ***y***_*Bj*_ plus the individual deviations from the group average ***y***_*Wij*_:

(1) $$y_{ij}=y_{Bj}+y_{Wij}$$

where the unobserved random components ***y***_*Bj*_ and ***y***_*Wij*_ are assumed to be independent with expected values *E*(***y***_*Bj*_) = **μ** and *E*(***y***_*Wij*_) = 0 (cf. Muthén, 1994; Yuan and Bentler, 2007).

The measurement models for the endogenous random component vectors ***y***_*Bj*_ and ***y***_*Wij*_ are

(2) $$y_{Bj}=\mu +\Lambda_{B}^{y}\eta_{Bj}+\varepsilon_{Bj},y_{Wij}=\Lambda_{W}^{y}\eta_{Wij}+\varepsilon_{Wij}$$

where **μ** is a mean vector, **Λ**^*y*^_*B*_ and **Λ**^*y*^_*W*_ are the factor loading matrices, **η**_*B*_ and **η**_*W*_ are the latent criterion (dependent) variables, and **ε**_*B*_ and **ε**_*W*_ denote the residual vectors at the between and the within level. Analogously, the data vector ***x****_ij_* is also decomposed into two unobserved random component vectors, ***x***_*Bj*_ and ***x***_*Wij*_

(3) $$x_{ij}=x_{Bj}+x_{Wij}$$

where the vectors ***x***_*Bj*_ and ***x***_*Wij*_ are assumed to be independent with expected values *E*(***x***_*Bj*_) = **ν** and *E*(***x***_*Wij*_) = 0.

The measurement models for the exogenous random component vectors ***x***_*Bj*_ and ***x***_*Wij*_ are

(4) $$x_{Bj}=\nu +\Lambda_{B}^{x}\xi_{Bj}+\delta_{Bj},x_{Wij}=\Lambda_{W}^{x}\xi_{Wij}+\delta_{Wij}$$

where **ν** is a mean vector, **Λ**^*x*^_*B*_ and **Λ**^*x*^_*W*_ are the factor loading matrices, **ξ**_*B*_ and **ξ**_*W*_ are the vectors of latent predictor and moderator (independent) variables, and **δ***_B_* and **δ***_W_* denote the residual vectors at the between and the within level.

The unconstrained approach requires the forming of product indicators for defining the latent interaction terms. Although several alternative strategies exist for the construction of these indicators, most often used are the all-pair and the matched-pair strategies. Kenny and Judd (1984) as well as Jöreskog and Yang (1996) used the all-pair strategy for creating all possible cross-products to define the latent interaction term, while using the matched-pair strategy Marsh et al. (2004) showed that it is sufficient to use each indicator of the latent predictor and the moderator variable only once in forming the cross-products. As cross-products can be created by using different combinations of the indicators, Marsh et al. (2004) suggested matching the indicators by reliability as the interaction effects were found to be estimated with more precision when the indicators with the highest factor loadings were matched to form the cross-products. The matched-pair strategy requires the number of indicators of the latent predictor and the latent moderator variable to be the same (for strategies using unequal numbers of indicators, cf. Jackman et al., 2011 and Wu et al., 2013).

When the structural models include predictor variable **ξ**_*B*_ and moderator variable **ξ**_*B*_ at the between level and predictor and moderator variables **ξ**_1*W*_ and **ξ**_2*W*_ at the within level, the measurement models for the latent interaction terms **ξ**_1*B*_**ξ**_2*B*_ and **ξ**_1*W*_**ξ**_2*W*_ are

(5) $$\begin{array}{l} \;x_{kBj}x_{lBj}=\tau_{B}+\Lambda_{B}^{xx}\xi_{1Bj}\xi_{2Bj}+\varsigma_{Bj},\\ x_{kWij}x_{lWij}=\tau_{W}+\Lambda_{W}^{xx}\xi_{1Wij}\xi_{2Wij}+\varsigma_{Wij} \end{array}$$

where **τ***_B_* and **τ***_W_* are mean vectors, **Λ**^*xx*^_*B*_ and **Λ**^*xx*^_*W*_ denote the factor loading matrices, ***x***_*kBj*_***x***_*lBj*_ are vectors of cross-products with *k* = 1,…, *K* random components as indicators of **ξ**_1_*_B_* and *l* = 1,…, *L* (*K* = *L*) random components as indicator variables of **ξ**_2*B*_, **ξ**_*kWij*_***x***_*lWij*_ are vectors of cross-products with *k* = 1,…, *K* random components as indicator variables of **ξ**_1*W*_ and *l* = 1,…, *L* (*K* = *L*) random components as indicator variables of **ξ**_2*W*_, and **ς**_*Bj*_ as well as **ς**_*Wij*_ denote the residual vector.

**Figure 1:**
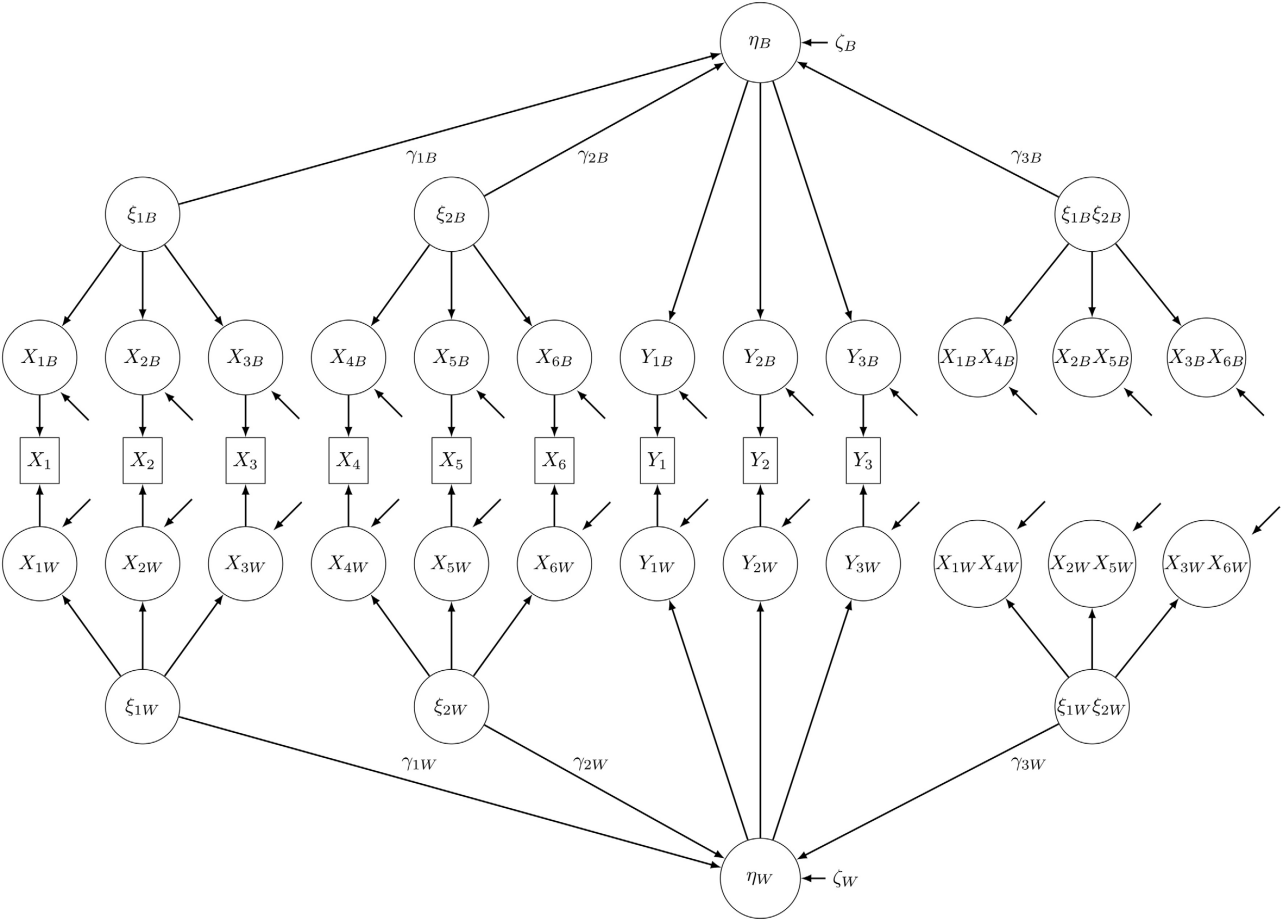
**Path diagram of the nonlinear population MSEM with latent interaction effects at both levels**. Product indicators were constructed using the matched-pairs strategy.

As nonlinear effects may occur at the between-group level, at the within-group level, or at both levels simultaneously, the structural equations for a model with two latent level-specific predictor variables (ξ_1_*_W_*, ξ_2_*_W_*), two moderator variables (ξ_1__*B*_, ξ_2_*_B_*), and a latent interaction term at both levels are then given by

(6) $$\begin{array}{l} \eta_{W}=\gamma_{1W}\xi_{1W}+\;\gamma_{2W}\xi_{2W}+\;\gamma_{3W}\xi_{1W}\xi_{2W}\\ \;+\zeta_{W}\text{ (within-group level)} \end{array}$$

(7) $$\begin{array}{l} \eta_{B}=\alpha +\gamma_{1B}\xi_{1B}+\;\gamma_{2B}\xi_{2B}+\;\gamma_{3B}\xi_{1B}\xi_{2B}+\\ \;\zeta_{B}\text{ (between-group level)} \end{array}$$

where α is the overall mean, γ_1*W*_, γ_2*W*_, and γ_3*W*_ are effects at the within level, γ_1*B*_, γ_2*B*_, and γ_3*B*_ are effects at the between level, and ζ_*W*_ and ζ_*B*_ are disturbance terms. In applied research, the between-group level predictors do not have to match the within-group level predictors, but in this study, we will only consider the model in Equations (6) and (7) (see also Figure 1).

Based on the decomposition in Equations (1) and (3) and under the assumption of identical covariance structures across groups and uncorrelatedness of within-group and between-group random components, the total covariance matrix Σ_*T*_ of the data vectors ***y*** and ***x*** is augmented by the cross-products, and the augmented total covariance matrix Σ^*^_T_ is then the sum of the between-group covariance matrix Σ^*^_B_ and the within-group covariance matrix Σ^*^_W_ (cf. Yuan and Bentler, 2007)

(8) $$Cov(y,\text{x})=\Sigma_{T}^{*}=\Sigma_{B}^{*}+\Sigma_{W}^{*}$$

where the asterisk denotes matrices augmented by product variables. The nonlinear MSEM therefore contains a covariance structure at each level augmented by matched-pairs of product variables, and these level-specific covariance matrices are needed for model fit evaluation.

## Model fit evaluation of nonlinear MSEM

### Overall model fit evaluation

For nonlinear MSEM, model fit evaluation is not as straightforward as it is for linear MSEM. For linear MSEM the standard procedure (Ryu and West, 2009) is often used which is based on the comparison of the unstructured covariance matrix with the model-implied covariance matrix of the entire model comparable to single-level SEM. An often used method for parameter estimation is the ML method. The ML fit function leads to a test statistic *T*_*ML*_ that is calculated as the product of the minimum of the fitting function *F*_*ML*_ and (*N* − 1), where *N* equals sample size. Under the assumptions of a correctly specified model, multivariate normally distributed variables, and a sufficiently large sample size, *T*_*ML*_ asymptotically follows a central χ^2^ distribution. A non-significant test statistic *T*_*ML*_ indicates that the model fits the data. The smaller the difference between both covariance matrices is the better the model fits the data.

The main problem with model fit evaluation for nonlinear MSEM is well-known from the evaluation of single-level nonlinear SEM: Model fit cannot be determined because a suitable saturated model does not exist (Klein and Schermelleh-Engel, 2010). For nonlinear SEM as well as for nonlinear MSEM the target model is not nested within the saturated model that is represented by the unstructured covariance matrix. The unstructured covariance matrix is not appropriate for model fit evaluation of nonlinear MSEM because covariances do not contain any information about the nonlinearity (e.g., interaction effects) in the data. For that reason, the assessment of overall model fit for a nonlinear SEM is still an unresolved problem.

Nevertheless, nonlinearity is contained in the product variables that form the measurement models of the latent product terms. The covariances between the product indicators and the *y*-variables are therefore indicative of existing nonlinear effects. For model fit evaluation the covariance matrix can therefore be augmented such that the new total covariance matrix Σ^*^_T_ comprises covariances between *y*-variables, *x-variables*, and the matched-pairs of cross-products of the *x*-variables.

For model fit evaluation, the likelihood ratio test of exact fit for nonlinear MSEM can then be performed which tests the hypothesis that both level-specific model-implied augmented covariance matrices are equal to their population matrices (cf. Ryu and West, 2009):

(9) $$\Sigma_{B}^{*}=\Sigma_{B}^{*}\left(\theta \right),\;\Sigma_{W}^{*}=\Sigma_{W}^{*}\left(\theta \right)$$

where θ is the parameter vector. For this omnibus test the ML test statistic based on the augmented covariance matrices can then be written as

(10) $$T_{ML}^{*}=F_{ML}\left[\Sigma_{W}^{*}\left(\hat{\theta }\right),\Sigma_{B}^{*}\left(\hat{\theta }\right)\right]-F_{ML}\left[\Sigma_{W}^{*}\left({\hat{\theta }}_{s}\right),\Sigma_{B}^{*}\left({\hat{\theta }}_{s}\right)\right]\;$$

where $\hat{\theta }$ denotes the vector of estimated parameters in the target model and $\hat{\theta }$_*s*_ denotes the vector of estimated parameters in the saturated model. Under the assumption of correctly specified models at both levels, multivariate normality and a sufficiently large number of groups, the test statistic follows a central χ^2^ distribution with *df*_*T*_ = *df*_*B*_ + *df*_*W*_ degrees of freedom.

Unfortunately, augmenting the empirical covariance matrix by product variables implies a multivariate nonnormal distribution. The reason is that products even of normally distributed variables are nonnormally distributed, i.e., highly kurtotic and often skewed (cf. Craig, 1936; Aroian, 1944; Moosbrugger et al., 1997; Klein and Moosbrugger, 2000). Therefore the assumption of multivariate normality of the ML estimation method is always violated when product terms are added to a structural equation model.

Depending on the strategy used for the construction of product terms, i.e., all-pair or matched-pair strategy, the amount of nonnormality in the data set differs. For example, if each latent predictor variable is measured by three indicators, for the all-pair strategy nine product terms have to be created, while for the matched-pair strategy only three product terms are needed. The all-pair strategy therefore produces a larger amount of nonnormality than the matched-pair strategy. If the covariance matrices are augmented by product variables due to the matched-pair strategy the amount of nonlinearity is kept to a minimum. Therefore the matched-pair strategy is used for the simulation study.

For model fit evaluation of linear MSEM it is recommended to use the rescaled test statistic of the ML estimator *T*_MLR_ when the normality assumption is violated (cf. Marsh et al., 2009; Hox, 2010; Kim et al., 2012). *T*_MLR_ adjusts the unscaled test statistic *T*_*ML*_ downward as a function of the multivariate kurtosis and may therefore correct the nonnormality due to highly kurtotic product variables sufficiently well. Robust test statistics are provided by several computer programs. In M*plus*, the robust test statistic *T*_MLR_ is provided which is asymptotically equivalent to the Yuan-Bentler T2^*^ test statistic (Muthén and Muthén, 1998–2012; see also Satorra and Bentler, 1994). In the simulation study we will use the rescaled test statistic based on the augmented covariance matrix T^*^_MLR_ and compare it to the uncorrected test statistic T^*^_ML_.

### Level-specific model fit evaluation

The standard approach for multilevel models evaluates the model fit for the entire model. However, this approach has some limitations (cf. Yuan and Bentler, 2007; Ryu and West, 2009). If both levels are evaluated simultaneously, a significant test statistic does not provide any information on the level at which the model is misspecified. Model misfit can exist at the between-group level, the within-group level or at both levels simultaneously. As sample size is typically much larger at the within-level than at the between-level, a much heavier weight is given to the within-group model fit than to the between-group model fit for calculating the overall fit statistic.

In order to deal with these problems two approaches exist. Yuan and Bentler (2007) proposed to use a segregating approach which fits the structural equation model at each level separately. They showed that model misfit can be detected satisfactorily and that the fit indices of single-level SEM can be extended to evaluating models at separate levels of a multilevel model. Ryu and West (2009, based on Hox, 2002) suggested to estimate partially saturated models. Model fit for one level is evaluated while the other level is specified as a saturated model. This approach showed quite similar results compared to Yuan and Bentler's (2007) segregating approach for the within-group model, but seemed to perform better with regard to a slightly lower non-convergence rate, a mean chi-square statistic closer to the nominal value, and a smaller Type I error rate for estimating the correct between-group model.

For evaluating the model fit of a nonlinear MSEM at the within-group level using the partially saturated approach, the within model is specified as the target model and the between model is specified as saturated. The test statistic for the partially saturated model is then

(11) $$T_{PS\_W}^{*}=F_{ML}\left[\Sigma_{W}^{*}\left(\hat{\theta }\right),\Sigma_{B}^{*}\left({\hat{\theta }}_{s}\right)\right]-F_{ML}\left[\Sigma_{W}^{*}\left({\hat{\theta }}_{s}\right),\Sigma_{B}^{*}\left({\hat{\theta }}_{s}\right)\right].$$

Any misfit at the within-group level is due to the discrepancy between Σ^*^_W_($\hat{\theta }$) and Σ^*^_W_($\hat{\theta }$_*s*_).

For evaluating the model fit of a nonlinear MSEM at the between-group level, the between model is specified as the target model and the within model as saturated. The test statistic is then obtained by

(12) $$T_{PS\_B}^{*}=F_{ML}\left[\Sigma_{W}^{*}\left({\hat{\theta }}_{s}\right),\Sigma_{B}^{*}\left(\hat{\theta }\right)\right]-F_{ML}\left[\Sigma_{W}^{*}\left({\hat{\theta }}_{s}\right),\Sigma_{B}^{*}\left({\hat{\theta }}_{s}\right)\right]$$

Any misfit at the between-group level is due to the discrepancy between Σ^*^_B_($\hat{\theta }$) and Σ^*^_B_($\hat{\theta }$_*s*_).

The degrees of freedom at both levels are calculated comparable to MSEM with linear effects as the difference between the number of parameters in the saturated model and the number of parameters in the target model. In addition to evaluating the complete model we will also evaluate partially saturated models in the simulation study.

## Methods

We conducted a Monte Carlo study with the aim of investigating the performance of the robust test statistic *T_MLR_* compared to *T_ML_* for nonlinear MSEM. As these test statistics are often also denoted as χ^2^ tests, we will use the terms χ^2^ test and robust χ^2^ test in the following. The model used for this study is a nonlinear MSEM with interaction effects at both levels (see Figure 1, see also Equations 6 and 7).

Using latent aggregation to account for sampling error the manifest indicators of the latent variables were split into their latent within and between components (see Figure 1). Latent aggregation is the default option in M*plus* for treating within and between components as latent unobserved covariates (Asparouhov and Muthén, 2007). Using the FSCORES option in M*plus* the estimated values of the latent components at the between-group level, ***x_Bj_***, were obtained from random intercept models. In the next step, the estimated values of the latent components at the within-group level, ***x_Wij_***, were calculated by simple subtraction. The vectors ***x_Wij_*** and ***x_Bj_*** can be regarded as latent within and between components of the manifest indicator variables ***x**_ij_* of the latent predictor variables. Finally, products of the within and between components, *x*_1*W*_ *x*_4*W*_, …, *x*_3*B*_*x*_6*B*_ (see Figure 1), were calculated using the matched-pair strategy.

Data for four population models (M) with different numbers of nonlinear effects were generated: (1) a model with linear effects at the within-group level (W) and the between-group level (B), but no nonlinear effects (0) at either levels (M_W0B0), (2) a model with an interaction effect (I) only at the within-group level (M_WIB0), (3) a model with an interaction effect only at the between-group level (M_W0BI), and (4) a model with interaction effects at both levels (M_WIBI).

Depending on the model, population parameters γ_3*W*_ (within-group level) and γ_3*B*_ (between-group level) were set to either 0 or to 0.20. The indicators each had a reliability of 0.80. This resulted in factor loadings of 1.00 for the scaling variables and 0.894 for all other indicators. Accordingly, error variances were 0.25 for the scaling variables and 0.20 for all other indicators. The linear effects γ_1*W*_, γ_2*W*_, and γ_1*B*_, γ_2*B*_ were set to 0.30. The population mean of η_*B*_ was set to zero by selecting the intercept α accordingly. The variances of the latent dependent variables η_*W*_ and η_*B*_ were set to 1.0, and the variances of the latent residuals ζ_*W*_ and ζ_*B*_ were selected accordingly with values between 0.82 (model with no interaction effects) and 0.72 (model with interaction effects and correlated predictors). Since population values at the within- and between-group level were set equal, the intra class correlation coefficient was 0.50 for all manifest variables. These parameter values were held constant across all simulations.

The latent predictor variables at the between-group level and the within-group level, ξ_1*B*_, ξ_2*B*_, and ξ_1*W*_, ξ_2*W*_, as well as all residual variables were generated as multivariate normally distributed variables.

### Simulation conditions

In the simulation study, three factors were varied: number of groups (three levels: *NG* = 200, 500, 1000), correlation of latent exogenous variables (two levels: ϕ_21_ = *Corr*(ξ_1*W*_, ξ_2*W*_) = *Corr*(ξ_1*B*_, ξ_2*B*_) = 0, 0.30), and the number of nonlinear effects in the complete population model (four levels: no interaction effects, within-group interaction effect, between-group interaction effect, and interaction effects at both levels). The total number of conditions was therefore 3 × 2 × 4 = 24. The numbers of groups were selected to ensure convergence. Prestudies, not reported here, indicated estimation problems using less than 200 groups. The number of subjects (*NS*) in each group was set to 30 to achieve a balanced design. Fixing the sample size of each group at *N* = 30 yielded total sample sizes of 6000, 15,000, and 30,000 subjects, respectively. The value for the latent predictor covariance ϕ_21_ was either 0 or 0.30. The amount of explained variance of the endogenous latent variables varied for the population models between 18 and 28%.

For each condition 500 datasets were generated using the statistical software R (R Core Team, 2013), and each dataset was analyzed using the program M*plus*, version 7 (Muthén and Muthén, 1998–2012).

### Analysis models

Using the ML and the MLR estimation method, overall model fit was evaluated for complete multilevel models and for partially saturated models (see Table 1). Model fit of partially saturated models was evaluated level-specific by saturating one level and analyzing the other level, while the model fit for both levels was estimated by simultaneously analyzing complete models not saturated at any level. Model misspecification at one level or at both levels was either established by fixing the (existing) interaction effect to zero while keeping the latent interaction term in the structural equation, or by including the (nonexistent) interaction effect in the model.

**Table 1:** **Overview over analysis models used for overall model fit evaluation by means of χ^2^ tests and for evaluation of single interaction effects by means of χ^2^ difference tests for complete MSEM models and for partially saturated models (PS) at the within-group (W_*s*_) or at the between-group (B_*s*_) level**.

**Population model**	**Type I error**	**Power**
**χ^2^ TEST**		
M_WIBI	WIBI	*W0*BI
	PS_WIB_*s*_	WI*B0*
	PS_W_*s*_BI	PS_*W0*B_*s*_
		PS_W_*s*_*B0*
**χ^2^ DIFFERENCE TEST**		
M_W0B0	W0B0 vs. *WIBI*	
	PS_W0B_*s*_ vs. PS_*WI*B_*s*_	
	PS_W_*s*_B0 vs. PS_W_*s*_*BI*	
M_WIB0		*W0*B0 vs. WIB0
		PS_*W0*B_*s*_ vs. PS_WIB_*s*_
M_W0BI		W0*B0* vs. W0BI
		PS_W_*s*_*B0* vs. PS_W_*s*_BI

Population models are denoted by M, analysis models are denoted by their respective within- or between-levels (W, B), I indicates an interaction effect at the within- or between-group level (WI, BI), 0 indicates a missing interaction effect (W0, B0), PS are partially saturated analysis models at the within- or between-level (W_*s*_, B_*s*_). Misspecified models with either an interaction effect added to a linear model or an existing interaction effect fixed to zero are in italics.

As the misspecified models and the correctly specified models are nested, it was also possible to conduct χ^2^ difference tests for the evaluation of single nonlinear effects. While the overall χ^2^ statistic tests all restrictions in the model simultaneously, the χ^2^ difference statistic only tests the significance of single parameters. The model difference test is generally preferred to the *t*-test, as standard errors of the *t*-test are known to be biased when the assumption of multivariate normality is violated. In order to determine the power of the χ^2^ difference tests as well as their Type I error rates, the unscaled χ^2^ difference value and not the scaled χ^2^ difference value proposed by Satorra and Bentler (2001) was used for nested model comparisons as suggested by Gerhard et al. (in press) and Cham et al. (2012) for nonlinear SEM. Nested model difference tests were performed for complete as well as for partially saturated models (cf. Table 1).

In the following, the analysis models are denoted comparable to the population models (see Table 1) but without the “M” at the beginning of the name: “W” and “B” again indicate models at the within- and the between-group level, “I” indicates that an interaction effect is present while “0” indicates that the nonlinear effect is fixed to zero.

There were four different types of analysis models: (1) models estimating linear effects at both levels while the interaction effects were fixed to zero (W0B0); (2) models estimating an interaction effect at the within-group level but no interaction effect at the between-group level (WIB0); (3) models estimating an interaction effect at the between-group level but not at the within-group level (W0BI), and (4) models estimating interaction effects at both levels (WIBI). Additionally, there were two types of partially saturated analysis models: Either the between-group level was saturated (B_*s*_) while model fit at the within-group level was evaluated (PS_W0B_*s*_, PS_WIB_*s*_), or the within-group level was saturated (W_*s*_) while model fit at the between-group level was evaluated (PS_W_*s*_B0, PS_W_*s*_BI).

### Evaluation of the model tests

For all types of analysis models the means of the standard χ^2^ values and the means of the robust χ^2^ values for estimating overall model fit were obtained from 500 replications for each condition, and the rejection rates at the nominal level of α = 0.05 were computed. The rejection rates for linear models with interaction effects additionally included in the model can be interpreted as the Type I error of the χ^2^ test, the rejection rates for misspecified models with interaction effects fixed to zero can be interpreted as the power of the χ^2^ test to detect misspecification. Analogously, means of χ^2^ difference values, Type I error rates, and power for χ^2^ difference tests were obtained.

## Results

In the following, we will only report a representative selection of the different analyses (see Table 1) because the results not reported here lead to similar conclusions. No non-convergent or inadmissible solutions (e.g., negative variance estimates) were encountered across all simulated data sets. First, mean χ^2^ values and Type I error rates for the overall model test by comparing the ML and MLR estimators are given in Table 2. As MLR outperformed ML in all conditions, only MLR results are reported in the subsequent Tables. Power rates for misspecified models at the within-group level and at the between-group level are given in Table 3. Second, results of the χ^2^ difference tests include Type I error rates (Table 4), power rates at the within-group level (Table 5), and power rates at the between-group level (Table 6).

**Table 2:** **ML and MLR mean χ^2^ values of overall model fit and Type I error rates for the population model with interaction effects at both levels (M_WIBI) analyzed with the correct complete model (WIBI), the correct partially saturated within model (PS_WIB_*s*_), and the correct partially saturated between model (PS_W_*s*_BI) under conditions of varying numbers of groups (*NG*) and uncorrelated predictor variables (Φ_21_ = 0)**.

**Population model: M_WIBI**									
				**Analysis models**					
	**WIBI**			**PS_WIB**_s_****			**PS_W**_s_**BI**		
***NG***	**χ^2^**	***df***	**Type I error (%)**	**χ^2^**	***df***	**Type I error (%)**	**χ^2^**	***df***	**Type I error (%)**
**ML**									
200	113.59	100	25.6	56.74	50	18.2	56.87	50	17.4
500	113.27	100	26.6	56.63	50	17.2	56.65	50	18.2
1000	111.65	100	20.4	55.75	50	15.0	55.89	50	15.6
**MLR**									
200	99.65	100	6.0	50.72	50	7.8	49.03	50	5.6
500	97.30	100	3.6	50.48	50	7.2	46.97	50	3.4
1000	95.20	100	2.8	49.65	50	4.2	45.75	50	2.6

χ^2^ is the mean of the Monte Carlo χ^2^ values.

**Table 3:** **MLR mean χ^2^ values of overall model fit and power rates for the population model with interaction effects at both levels (M_WIBI) analyzed with complete models misspecified at the within level (*W0*BI) or at the between level (WI*B0*), and analyzed with partially saturated models with misspecification at the within level (PS_*W0*B_*s*_) or the between level (PS_W_*s*_*B0*) under conditions of varying numbers of groups (NG) and correlation of predictor variables (Φ_21_)**.

**Population model: M_WIBI**						
**MISSPECIFIED ANALYSIS MODELS AT THE WITHIN-GROUP LEVEL**						
	***W0*BI**			**PS_*W0*B**_s_****		
**NG**	**χ^2^**	***df***	**Power (%)**	**χ^2^**	***df***	**Power (%)**
**Φ**_21_** = **0****						
200	278.53	101	100	233.32	51	100
500	534.43	101	100	504.91	51	100
1000	966.16	101	100	960.51	51	100
**Φ**_21_** = **0.30****						
200	314.09	101	100	268.44	51	100
500	613.68	101	100	588.01	51	100
1000	1117.46	101	100	1118.05	51	100
**MISSPECIFIED ANALYSIS MODELS AT THE BETWEEN-GROUP LEVEL**						
	**WI*B0***			**PS_W_*s*_*B0***		
**NG**	**χ^2^**	***df***	**Power (%)**	**χ^2^**	***df***	**Power (%)**
**Φ**_21_** = **0****						
200	106.69	101	12.0	55.98	51	13.4
500	112.66	101	21.6	61.87	51	28.8
1000	125.29	101	49.4	74.72	51	66.6
**Φ**_21_** = **0.30****						
200	108.39	101	13.2	58.03	51	16.0
500	113.69	101	20.8	63.68	51	30.4
1000	130.93	101	57.8	80.45	51	77.0

χ*^2^* is the mean of the Monte Carlo χ *^2^* values. Misspecified models with either an interaction effect added to a linear model or an existing interaction effect fixed to zero are in italics.

**Table 4:** **MLR mean χ^2^ difference values (Δχ^2^) and Type I error rates for comparing correctly specified complete or partially saturated models without nonlinear effects (W0B0, W0Bs, WsB0) with misspecified models with an added interaction effect at both levels, at the within-group level (*WI*), or at the between-group level (*BI*) under conditions of varying numbers of groups (NG) and varying correlation of predictor variables (Φ_21_)**.

**Population model: M_W0B0**									
	**W0B0 vs. *WIBI***			**PS_W0B**_s_** vs. PS_*WI*B**_s_****			**PS_W**_s_**B0 vs. PS_W**_s_***BI***		
**NG**	**Δ χ^2^**	**Δ *df***	**Type I error (%)**	**Δ χ^2^**	**Δ *df***	**Type I error (%)**	**Δ χ^2^**	**Δ *df***	**Type I error (%)**
**Φ**_21_** = **0****									
200	2.05	2	3.4	1.00	1	3.8	1.05	1	3.6
500	1.94	2	3.4	0.93	1	3.4	1.01	1	3.4
1000	2.07	2	4.6	1.05	1	4.4	1.02	1	3.6
**Φ**_21_** = **0.30****									
200	2.23	2	5.4	1.05	1	5.0	1.18	1	5.6
500	2.22	2	6.0	1.11	1	6.0	1.11	1	3.8
1000	1.99	2	3.6	1.07	1	4.6	0.91	1	3.2

Δχ^2^ is the mean of the Monte Carlo χ ^2^ difference values. Misspecified models with either an interaction effect added to a linear model or an existing interaction effect fixed to zero are in italics.

**Table 5:** **MLR mean χ^2^ difference values (Δχ^2^) and power rates for comparing misspecified complete or partially saturated models with a fixed interaction effect at the within-group level (*W0*) with the correct models (WIB0, PS_WIBs) without nonlinear effects at the between-group level under conditions of varying numbers of groups (NG) and varying correlation of predictor variables (Φ_21_)**.

**Population model: M_WIB0**						
	**Misspecified Analysis Models at the Within-Group Level**					
	***W0*B0 vs. WIB0**			**PS_*W0*B**_s_** vs. PS_WIB_s_**		
**NG**	**Δχ^2^**	**Δ*df***	**Power (%)**	**Δχ^2^**	**Δ*df***	**Power (%)**
**Φ_21_ = **0****						
200	180.22	1	100	183.61	1	100
500	439.28	1	100	455.09	1	100
1000	872.00	1	100	909.39	1	100
**Φ_21_ = **0.30****						
200	212.27	1	100	215.98	1	100
500	518.49	1	100	536.94	1	100
1000	1026.03	1	100	1070.55	1	100

Δ χ^2^ is the mean of the Monte Carlo χ^2^ difference values. Misspecified models with either an interaction effect added to a linear model or an existing interaction effect fixed to zero are in italics.

**Table 6:** **χ^2^ difference mean values (Δχ^2^) and power rates for comparing misspecified complete or partially saturated models with a fixed interaction effect at the between-group level (*B0*) with the correct models (WIB0, PS_WsBI) without nonlinear effects at the within-group level under conditions of varying numbers of groups (NG) and varying correlation of predictor variables (Φ_21_)**.

**Population model: M_W0BI**						
	**Misspecified Analysis Models at the Between-Group Level**					
	**W0*B0* vs. W0BI**			**PS_W_s_*B0* vs. PS_W_s_BI**		
**NG**	**Δχ^2^**	**Δ*df***	**Power (%)**	**Δχ^2^**	**Δ*df***	**Power (%)**
**Φ**_21_** = **0****						
200	7.04	1	68.0	6.93	1	68.0
500	15.11	1	96.4	14.63	1	96.0
1000	29.48	1	100	28.35	1	100
**Φ**_21_** = **0.30****						
200	7.95	1	75.4	7.86	1	74.6
500	18.05	1	98.6	17.51	1	98.6
1000	35.36	1	100	34.04	1	100

Δ χ^2^ is the mean of the Monte Carlo χ^2^ difference values. Misspecified models with either an interaction effect added to a linear model or an existing interaction effect fixed to zero are in italics.

### Overall model fit

MLR mean χ^2^ values and Type I error rates for the population model M_WIBI are given in Table 2. For the ML estimator Type I error rates were inflated across all conditions, independent of the level being analyzed. The difference between observed mean χ^2^ values and degrees of freedom was higher for the complete model (WIBI) than for the partially saturated models (PS_WIB_*s*_, PS_W_*s*_BI). Type I error rates using the MLR estimator for the population model with interaction effects at both levels (M_WIBI) were close to the nominal α level with values ranging between 2.6 and 7.8%. Results also indicated that the MLR estimator showed a slightly more conservative behavior for higher numbers of groups (*NG* = 500, 1000) when the models partially saturated at the within-group level were analyzed.

MLR mean χ^2^ values and power rates for the population model M_WIBI are listed in Table 3. The results show that model misspecifications at the within-group level could be reliably detected. When the within-group interaction effect in the analysis models was fixed to zero, high χ^2^ values indicated significant model misfit, and the rejection rate was 100% across conditions. Mean χ^2^ values ranged from 278 to 1117 for the analyses of the complete model with misspecification at the within-group level (*W0*BI) with higher values in conditions with a larger number of groups. The χ^2^/*df*-ratio was larger for partially saturated models (PS_*W0*B_*s*_) than for the unsaturated models.

Model misspecification at the between-group level with the interaction effect fixed to zero was less reliably detected (see Table 3). The rejection rates ranged from 12 to 58% for the complete model (WI*B0*) and from 13 to 77% for the partially saturated model (PS_W_*s*_*B0*). Power rates were in all conditions higher in the partially saturated models than in the complete models and increased in conditions with higher numbers of groups and models, especially for models with correlated predictor variables.

For conditions with correlated predictor variables mean χ^2^ values and power rates were always larger than for conditions with uncorrelated predictors. However, these differences were relatively small.

### χ^2^ difference tests

In order to investigate the behavior of the model difference test for detecting single interaction effects at one level or at both levels simultaneously, several model comparisons were performed. MLR mean χ^2^ difference values and Type I error rates for the comparison of the correctly specified model without interaction effects at both levels (M_W0B0) with misspecified models which additionally included an interaction effect either at both levels simultaneously, or at the within-group level or the between-group level only, are listed in Table 4. The results show that Type I error rates were close to the nominal α level and tended to be a bit conservative in conditions with uncorrelated predictor variables. Results of partially saturated models did not deviate from the results of complete models, and Type I error rates did not depend on the number of groups.

In Tables 5 and 6, MLR mean χ^2^ difference values and power rates for population models M_WIB0 and M_W0BI are listed. The results indicate that mean χ^2^ difference values were substantially higher for models with within-group misspecification than for models with between-group misspecification. Additionally, these values were considerably larger for increasing numbers of groups but only moderately larger for correlated predictor variables. Power of the χ^2^ difference test to detect within-group level misspecifications was 100% in all conditions (Table 5), while power to detect between-group level misspecifications ranged from 68 to 100% (Table 6).

## Discussion

In this study we investigated the overall model fit of MLR compared to ML for nonlinear MSEM with interaction effects at a single level or at both levels simultaneously. We also investigated χ^2^ difference tests for detecting single interaction effects. The core findings are:

1. MLR corrected the overall test statistic sufficiently well, while ML always yielded inflated χ^2^ values. Therefore only MLR results were reported.
2. For properly specified models, Type-I error rates of the χ^2^ test were close to their nominal α-levels.
3. Misspecification at the within-group level was reliably detected using the χ^2^ test, while the power to detect misspecification at the between-group level was fairly low.
4. The MLR χ^2^ difference test performed generally fairly well with regard to Type I error rates and power although for the smallest number of groups (*N* = 200) power of this test was low when models at the between-group level were analyzed compared to the within-group level.
5. Correlated predictors had a negligible effect such that the power to detect model misspecification slightly increased for both types of χ^2^ tests compared to models with uncorrelated predictors.

Although an adequate overall model test for nonlinear MSEM is not yet available, the likelihood ratio test based on covariance matrices augmented by product terms performed quite well. Using the robust test statistic *T_MLR_* of the M*plus* program, nonnormality resulting from nonlinearity in the model was corrected sufficiently well while *T*_*ML*_, which assumes multivariate normality, should not be used for model fit evaluation of nonlinear MSEM.

Compared to previous research (Yuan and Bentler, 2007; Ryu and West, 2009) the partially saturated approach was more informative than the standard approach. When model fit evaluation of the entire model indicated a poor fitting model, only level-specific evaluation was able to identify the specific level at which the misfit occurred. Power to detect misfit at the between-group level was quite low comparable to previous research (Ryu and West, 2009). Group sizes of *NG* = 200 did not seem to be sufficiently large to detect model misfit reliably, and even *NG* = 1000 resulted in low power for the standard approach (58%) and a power of 77% for the partially saturated approach for correlated latent exogenous variables. Power to detect misfit at the within-group level was always larger than power at the between-group level. This result could be expected because the total sample size was used for the analyses at the within-group level resulting in sample sizes up to 30,000 subjects.

Misspecified models were specified by fixing the nonlinear effects to zero while keeping the product indicators in the model. This type of misspecification is necessary for testing the significance of single nonlinear effects using a χ^2^ difference test, a test often used because it is generally more reliable than the *t*-test. In our simulation study Type I error rates of the overall χ^2^ test as well as the χ^2^ difference test were close to the nominal α level (see also Gerhard et al., in press). Power to detect misspecification of a single nonlinear effect was again larger at the within-group level than at the between-group level for both χ^2^ tests mirroring results of the partially saturated approach for ML-CFA (cf. Ryu and West, 2009).

As with all simulation studies there are some limitations which we would like to note. First, this study only considered a balanced design with within-group sample sizes held constant across groups. Additionally, the numbers of groups were large and the model structure and parameter values were identical at both levels. Further studies should investigate other designs which may be more appropriate for empirical research. Second, for the construction of the product indicators the matched-pair strategy was applied (Marsh et al., 2004) which uses each indicator of the latent exogenous constructs only once in specifying the cross-products. Alternatively, the all-pair strategy originally introduced by Kenny and Judd (1984) could have been applied which uses all possible cross-products. Using all possible products of indicator variables may be especially useful when the reliability of the indicator variables differs or when the number of indicators of the latent predictor and moderator variable are unequal. In our example this would have resulted in nine instead of in three product indicators measuring each latent interaction term. Whether this amount of nonnormality introduced by the all-pair approach could be also corrected by MLR remains to be investigated in a later study. Third, the indicator variables were generated with zero means. Because we only used balanced designs, the grand mean was identical to the mean of the clusters and therefore multicollinearity could be reduced at both levels. As in applied research balanced designs are not to be expected, more research is needed in order to investigate the consequences of using different methods for centering variables in the context of nonlinear models.

In conclusion, the robust ML estimator performed quite well in reliably detecting misspecification of nonlinear MSEM. Although the results of our simulation study indicate that MLR corrects the test statistic sufficiently well especially at the within-group level when the unconstrained product indicator approach is used, further research is necessary in order to develop a model test which takes the specific type of nonnormality implied by latent nonlinear effects explicitly into account.

### Conflict of interest statement

The authors declare that the research was conducted in the absence of any commercial or financial relationships that could be construed as a potential conflict of interest.
